# A Novel Homozygous Missense Variant in the *LRRC32* Gene Is Associated With a New Syndrome of Cleft Palate, Progressive Vitreoretinopathy, Growth Retardation, and Developmental Delay

**DOI:** 10.3389/fped.2022.859034

**Published:** 2022-05-17

**Authors:** Zufit Hexner-Erlichman, Boris Fichtman, Yoav Zehavi, Morad Khayat, Haneen Jabaly-Habib, Lee S. Izhaki-Tavor, Moshe Dessau, Orly Elpeleg, Ronen Spiegel

**Affiliations:** ^1^Department of Pediatrics, Emek Medical Center, Afula, Israel; ^2^Azrieli Faculty of Medicine, Bar-Ilan University, Safed, Israel; ^3^Rappaport School of Medicine, Technion - Israel Institute of Technology, Haifa, Israel; ^4^Genetic Institute, Emek Medical Center, Afula, Israel; ^5^Department of Ophthalmology, Baruch Padeh Medical Center, Poriya, Israel; ^6^Department of Genetics, Hadassah-Hebrew University Medical Center, Jerusalem, Israel

**Keywords:** cleft palate, retinopathy, TGF-β, *LRRC32* gene, GARP protein

## Abstract

Cleft lip and/or cleft palate are a common group of birth defects that further classify into syndromic and non-syndromic forms. The syndromic forms are usually accompanied by additional physical or cognitive abnormalities. Isolated cleft palate syndromes are less common; however, they are associated with a variety of congenital malformations and generally have an underlying genetic etiology. A single report in 2019 described a novel syndrome in three individuals, characterized by cleft palate, developmental delay and proliferative retinopathy due to a homozygous non-sense mutation in the *LRRC32* gene encoding glycoprotein A repetitions predominant (GARP), a cell surface polypeptide crucial for the processing and maturation of transforming growth factor β (TGF-β). We describe a patient who presented with cleft palate, prenatal and postnatal severe growth retardation, global developmental delay, dysmorphic facial features and progressive vitreoretinopathy. Whole exome sequencing (WES) revealed a very rare homozygous missense variant in the *LRRC32* gene, which resulted in substitution of a highly conserved isoleucine to threonine. Protein modeling suggested this variant may negatively affect GARP function on latent TGF-β activation. In summary, our report further expands the clinical features of cleft palate, proliferative retinopathy and developmental delay syndrome and emphasizes the association of *LRRC32* pathogenic variants with this new syndrome.

## Introduction

Cleft lip and/or cleft palate (CL/P) are the most frequent craniofacial birth defects.

CL/P is generally classified into syndromic and non-syndromic forms. Non-syndromic CL/P comprise 93–95% of the cases, display non-specific malformations and usually have multifactorial etiology, and presumably result from interaction between genetic susceptibility and environmental factors. Syndromic forms comprise 5–7% of all cases and are characterized by additional physical or cognitive abnormalities (1). More than 250 syndromes in which clefting is a primary feature are known to be caused by a mutation in a single genetic locus or chromosomal abnormality. Of those, Van der Woude syndrome, the most common form of syndromic CL/P, accounts for 2% of the cases. This syndrome is caused by autosomal dominant pathogenic variants in the interferon regulatory factor 6 gene *IRF6* (2, 3).

Isolated cleft palate is the rarest form of oral clefting and can present as syndromic or non-syndromic. The syndromic forms are associated with additional structural abnormalities, of which the most frequent are congenital heart defects, hydrocephalus, urinary tract malformations and polydactyly; and are commonly caused by an underlying well-defined genetic defect (4). A single report, published in 2019, described three children with a novel autosomal recessive syndrome, characterized by cleft palate, developmental delay and proliferative retinopathy (OMIM # 619074) due to a single homozygous non-sense mutation in the *LRRC32* gene (5). To our knowledge, no additional patients with this syndrome have been reported since.

In this report, we describe a patient with a novel homozygous missense variant in the *LRRC32* gene who presented with cleft palate, prenatal and postnatal severe growth delay, global developmental delay, dysmorphic facial features and progressive vitreoretinopathy. In addition, we summarize the current clinical and genetic characteristics of the new *LRRC32* associated syndrome.

## Case Report

The patient is a 15-year-old male, the second offspring of healthy first-degree cousins of Arab Muslim origin. His five siblings are healthy. His pregnancy was remarkable for symmetric intrauterine growth restriction. He was born prematurely at 34 weeks gestation following Cesarean section due to fetal distress and placental abruption. His birth weight was 1,170 g (2nd centile), length 38 cm (–3 *SD* below the mean) and head circumference 27 cm (–3 *SD* below the mean). His course at the neonatal intensive care unit was complicated by grade I retinopathy of prematurity, grade I intraventricular hemorrhage and severe necrotizing enterocolitis. The latter necessitated abdominal surgery with installation of an ileostomy, which was surgically closed at age 4 months. Additionally, he had cleft palate that was corrected surgically at age 1 year. His first 2 years of life were marked by severe failure to thrive, with growth indices (weight and height) ranging 3–4 standard deviations below the mean; microcephaly with dysmorphic facial features including triangular face, micrognathia, posteriorly rotated ears, a high protruding nasal bridge; and mild to moderate global developmental delay. Brain magnetic resonance imaging (MRI) at age 2 years was consistent with the previously known right parietal and temporal lobes premature hemorrhage. At age 2 years, the patient developed severe progressive dilated cardiomyopathy, with enlarged left ventricle and severely reduced shortening fraction of 11%. This necessitated maximal drug therapy including angiotensin-converting enzyme inhibitor, diuretics and digoxin. His cardiomyopathy progressed further during febrile illnesses and resulted in significant heart failure, suggesting metabolic etiology. Hence, metabolic investigations were performed, which demonstrated severely reduced serum-free carnitine levels, compatible with primary carnitine deficiency. This was confirmed by genetic analysis, which identified the homozygous p.Glu452Lys (c.1354 G > A) mutation in the *SLC22A5* gene, encoding the carnitine transporter. Carnitine supplementation, initially at 300 mg/kg/day resulted in considerable improvement of his cardiac malfunction. Within 12 months, the patient achieved normal heart function, enabling complete discontinuation of cardiac medications. A follow-up brain MRI at age 9 years showed extended T1 relaxation in the right temporal and parietal lobes, and significant white matter atrophy in the periventricular and subcortical areas.

Despite the carnitine deficiency that was appropriately treated, the patient displayed a phenotype that was unexplained by the primary carnitine deficiency. This included significant growth delay, mild to moderate intellectual disability, repaired cleft palate and facial dysmorphic features, as described.

**FIGURE 1:**
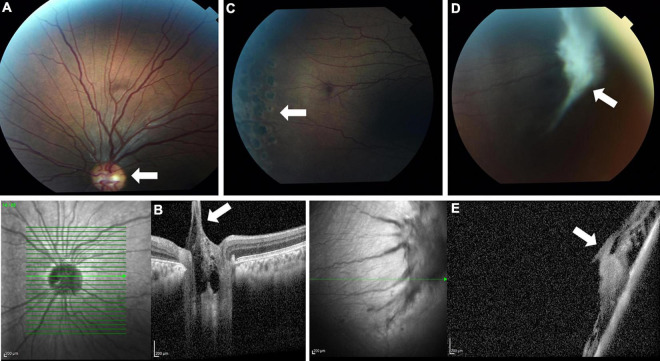
Fundus photo **(A)** and optical coherence tomography (OCT) **(B)** showing Bergmeister’s papillae and straightened retinal vessels (white arrows). Laser photocoagulation scars are seen (arrow) on fundus examination **(C)**. A later fundus image **(D)** and OCT **(E)** show a large new vitreoretinal traction (white arrows).

Ophthalmological evaluation at age 13 years demonstrated mildly decreased visual acuity of 20/30 in the right eye and 20/40 in the left eye. His ocular movements and his anterior segment examination were normal. Fundoscopic examination showed bilateral pink optic discs with Bergmeister’s papillae (tufts of fibrous tissue that indicate a remnant of hyaloid artery, which is usually completely regressed before birth) and straightened retinal vessels (Figure 1A). These retinal abnormalities were also evident by optical coherence tomography, which revealed retinopathy mainly involving the vitreal regions (Figure 1B). A thickened vitreous firmly attached to the retinal periphery was noted, with traction and secondary retinal tears. The patient underwent preventive argon laser photocoagulation in both eyes (Figure 1C). One year later, on follow up examination, a new large vitreoretinal traction on the peripheral retinal region of the left eye was seen (Figures 1D,E). A second laser photocoagulation treatment was done to prevent retinal detachment. Taken together, the ophthalmological findings are consistent with early onset progressive vitreoretinopathy.

## Materials and Methods

The study was approved by the Emek Medical Center ethics committee (study no. EMC-0067-09). Informed consent for participation in the study was obtained from all individual participants included in the study or their legally authorized representative (parents).

### Whole Exome Sequencing

Exonic sequences were enriched in DNA samples of the patient and both his parents (“trio exome”) using SureSelect Human All Exon ∼51 Mb Kit V5 (Agilent Technologies, Santa Clara, California, United States). Sequences were determined by HiSeq2500 (Illumina, San Diego, California, United States) as 125-bp, and were read paired-end. Read alignment and variant calling were performed with DNAnexus (Palo Alto, California, United States) using the default parameters with the human genome assembly hg19 (GRCh37) as a reference.

### Sanger Sequencing

Sequence analysis using genomic DNA from the patient, healthy siblings, and their parents was performed by amplification of a 422 bp fragment of exon 3 of LRRC32, containing the variant identified through exome sequencing. The sense 5′-TACCTGAACTTGTCCAACAA-3′ and the antisense 5′-AGATTGGCAAAGGTGTATGG-3′ primers were used under the following PCR conditions for DNA amplification: denaturation at 94^°^C for 5 min; 35 subsequent amplification cycles performed at 94^°^C for 30 sec, at 55^°^C for 45 sec and at 72^°^C for 30 s; and at 72^°^C for 5 min. The sequencing reaction was performed using the Bigdye terminator kit and analyzed by the 3500xl Genetic Analyzer (Applied Biosystems, Warrington, United Kingdom), according to the manufacturer’s instructions.

### Structural Analysis

The crystal structure of GARP_ECD_:TGF-β1:MHG-8_Fab_ (PDB ID 6GFF) was used as a model to evaluate the effect of the p.Ile327 > Thr variant on GARP protein structure and function. To illustrate the I327T variant in GARP’s ectodomain, we utilized the PyMOL mutagenesis tool to generate a structural model for this site. All hydrophobicity calculations and presentations were carried out using the PyMOL molecular graphics system (Version 2.0 Schrödinger, LLC).

## Results

### Molecular Genetic Analysis

**FIGURE 2:**
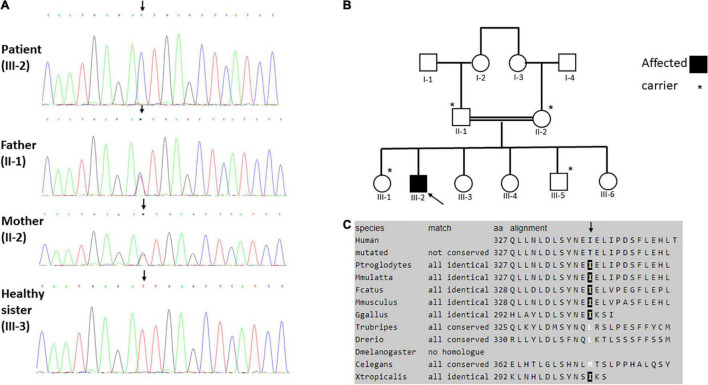
**(A)** Sanger sequencing showing the *LRRC32* homozygous c.980T > C variant in the patient (upper panel), the heterozygous variant in the healthy carrier father and mother (middle panels) and healthy non-carrier sister (lower panel). **(B)** The family pedigree, illustrating the c.980T > C variant state in all five of the patient’s siblings. **(C)** Evolutionary conservation of Ile327 (arrow). *means carrier (heterozygous).

Whole exome sequencing (WES) analysis for the patient and his parents was initiated after obtaining the relevant written informed consents and local ethical review board approval. Exome analysis of the proband yielded 59 million reads, with an average coverage of 80X. Following alignment and variant calling, we performed a series of filtering steps. These included removing variants that were called less than X8 or were off-target (>8 bp from splice junction), synonymous or had minor allele frequency (MAF)>0.005 at the Genome Aggregation Database (GnomAD browser).^1^ The analysis identified the previously reported homozygous pathogenic variant c.1354 G > A in the *SLC22A5* gene, which is known to cause a primary carnitine deficiency (6). In addition, we identified the homozygous c.980T > C variant in the *LRRC32* gene, which was not found in known databases. This was a different homozygous variant than was recently associated with a similar phenotype as our patient (5). Our variant was confirmed by Sanger sequencing and was submitted to ClinVar database (SUB10890028). The variant was inherited from both parents and family segregation confirmed that the variant was not found in the homozygous state in any of the patient’s five phenotypically normal siblings. Two siblings (III-1 and III-5) were heterozygous and three siblings (III-3, III-4, and III-6) did not carry the variant (Figures 2A,B). We were unable to study grandparents of the patient for their carrier state.

### Structural Analysis

**FIGURE 3:**
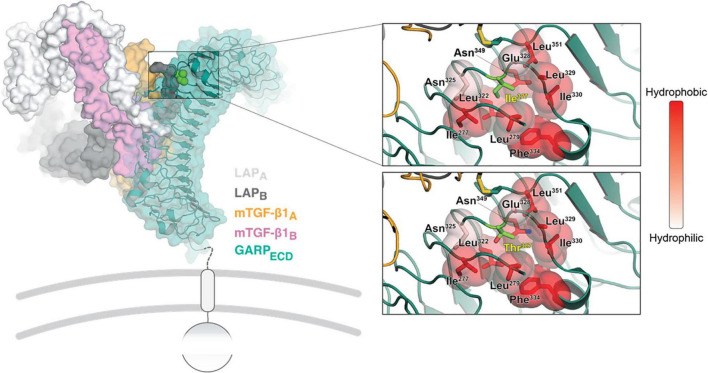
Structural analysis of the GARP Ile327 > Thr mutation. On the left, an overview is shown of the crystal structure of GARP in complex with TGF-β1 and stabilizing antibody MHG-8_Fab_ (PDB ID 6GFF). The top right close-up window shows the Ile327 site. The surrounding residues are colored in shades of red according to their hydrophobicity (right bar). The same site is shown with the Ile327 substitute and with a Thr residue right bottom). The local backbone and surrounding residues were energy minimized using the PyMol build tool.

The *LRRC32* gene encodes GARP, a transmembrane protein, retained at the surface of various cell types (7–9). The extracellular domain of GARP (GARP_ECD_) adopts a solenoid horseshoe structure, featuring 20 leucine-rich repeats (R1 to R20) capped by N- and C-terminal motifs, as was observed on the crystal structure of GARP in complex with two copies of the latency associated peptide, LAP (10) (Figure 3, left). The c.980T > C variant results in a threonine residue in position 327 instead of isoleucine (Ile327). Ile327 is located between leucine-rich repeat R11 and R12, and is adjacent to Cys350. The latter is involved in one of the two intermolecular disulfide bonds between GARP and each of the LAP molecules (10). Substitution of isoleucine to threonine has a double impact on the stability of that region of the protein, which can destabilize the whole structure. The Ile327 residue faces a hydrophobic cavity formed by the surrounding residues. Introducing a threonine residue (a polar residue) at that position causes repulsion between Thr327, Ile277 and Leu322, destabilizing the LRR10-11 (Figure 3, right panel). Moreover, the smaller residue of threonine has other implications on GARP’s integrity. Extensive investigations have shown an inverse ratio between protein stability and the volume of an unoccupied cavity in a protein’s core In our case, the Ile327 > Thr results in a larger unoccupied cavity, which forms due to the absence of the CD1 carbon atom that exists in Ile. This supports the plausibility that the stability of the p.Ile327 > Thr GARP variant may be compromised compared with the wild type (Figure 3, right panel).

## Discussion

Here we described a single patient who presented with cleft palate, global developmental delay, significant prenatal and postnatal growth retardation, severe dilated cardiomyopathy, proliferative vitreoretinal disease, microcephaly and dysmorphic features. This patient has two rare autosomal recessive diseases. Severe progressive dilated cardiomyopathy was identified already during infancy to be caused by primary carnitine deficiency. This was confirmed by biochemical assessment and genetic analysis that identified the previously reported c.1354 G > A homozygous mutation in the *SLC22A5* gene (6). However, the patient’s additional features were unexplained by this entity, suggesting a second underlying genetic defect. Thus, we employed trio exome sequencing in the patient and his parents. This analysis identified the homozygous novel c.980T > C variant in the *LRRC32* gene, which is consistent with a syndrome that has been reported in only three children, all from Israel (5).

**FIGURE 4:**
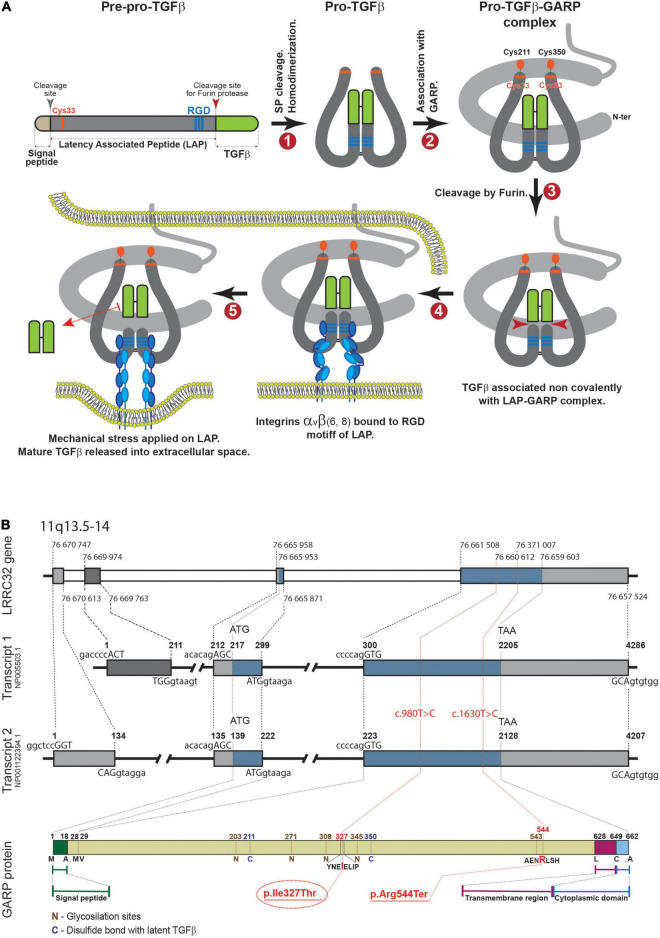
**(A)** Maturation process of TGF-β. **(B)** Upper panel. Genomic structure of *LRRC32*. Introns and exons are represented as gray and blue boxes, respectively. The start of each intron and exon is marked with dashed lines (nucleotide numbers are shown). Middle panel (transcripts 1, 2). Two major transcript types are shown. The numbers above each transcript delineate the beginning of each exon and intron, and the first nucleotides of start or stop codons. Sequences of 5′- and 3′-boundaries of exons are shown above and beneath each transcript, respectively (capital letters correspond to exons). In the lower panel, the protein product is shown with some key amino acids. The reported new variant is indicated in red.

The TGF-β superfamily consists of 33 proteins in mammals; of these, TGF-β is the prototype and most studied factor, displaying versatile functions in almost all cell types. As such, TGF-β is an evolutionary conserved pleiotropic factor that regulates many biological processes including development, cell proliferation and differentiation, tissue regeneration, stem cell and progenitor cell fates, apoptosis, and immune responses (11, 12). TGF-β is generated as a latent form, requiring subsequent binding to several molecules that enable activation in a highly tempospatially regulated manner (13, 14). GARP is a type 1 transmembrane protein encoded by the *LRRC32* gene. It is highly expressed by platelets, activated regulatory T cells, mesenchymal stromal cells and hepatic cells (15–17). It has a major role in the maturation course of TGF-β and thus it is associated in immune regulation, tumor progression and developmental processes (17, 18). Mature TGF-β (mTGF-β) is produced in the form of pre-pro-TGF-β, which consists of the N-terminal signal peptide (SP), latency associated peptide (LAP), and TGF-β at the C-terminal end of the polypeptide (19) (Figure 4A). Next, signal peptide removal and homodimerization by disulfide bonds are formed between two LAP-TGF-β peptides to form pro-TGF-β (step 1). The following step is the covalent association between the GARP protein, encoded by the *LRRC32* gene and pro-TGF-β *via* S-S bonds (Cys33 of TGF-β1, and Cys211 and Cys350 of GARP are involved) (step 2). Next, mTGF-β is cleaved from LAP by furin proteinase and is non-covalently associated with the LAP-GARP complex (step 3). The LAP-GARP-mTGFβ complex is then presented on the cell surface (step 4), where integrin avb6 or avb8 binds to the RGD motif on the LAP (15). Finally, active TGF-β is released into the extracellular space, presumably due to application of mechanical stress (16, 17) (Figure 4A).

The mTGF-β family plays a major role in palatogenesis, among other growth factors. This was nicely illustrated in TGF-β3 knockout mice who developed a cleft palate as a result of impaired adhesion of palatal shelves; and was also demonstrated *in vitro*, in mouse embryos using neutralizing antibody (18, 20, 21). Other members of the TGF-β superfamily were also shown to be involved in regulating epithelial mesenchymal transformation, mesenchymal cell proliferation and extracellular matrix synthesis in the palate (21, 22). Interruptions of these signaling pathways may lead to the formation of cleft palate.

Given the key role of GARP on TGF-β maturation, inherited defects in GARP are expected to result in TGF-β inactivation. Accordingly, garp knockout mice had early lethality and also defective palatogenesis (23).

In addition to the above, TGF-βs are expressed during periods of programmed cell death of some neuron populations, including in retinal development in chicks and mice. TGF-β2/TGF-β3 double deficient mice exhibit alterations in the cornea, lens and retina (24). Although scarce, these reports suggest a certain role of TGF-β activation in retinal development. Nevertheless, in the garp deficient murine model, retinal impairment was not observed (23). This may be explained by early lethality of the mice, which precludes later evolution of vitreoretinopathy.

Taken together, *LRRC32* loss of function variants result in GARP dysfunction and consequently abnormal maturation and inactivation of TGF-β. This may result in abnormal palate development such as cleft palate, and abnormal retinal development, given the substantial role of TGF-β in palatogenesis and retinal homeostasis. In accordance, three previously reported patients with the homozygous *LRRC32* p.Arg544Ter mutation (Figure 4B) presented a distinctive syndrome, which was highlighted by the presence of cleft palate and proliferative retinopathy. Similarly, our patient presented with cleft palate and later with childhood onset of proliferative vitreoretinopathy that was initially related to prematurity. However, the progressive nature and distinctive location of his retinopathy excluded retinopathy of prematurity as the cause of his ophthalmological disease. The phenotype displayed by our patient strikingly resembles the previously reported patients (Table 1). The missense *LRRC32* homozygous variant identified in our patient was not annotated in the ExAC and GnomAD databases and is predicted to change a highly evolutionary conserved isoleucine (Figure 2C). Additionally, *in silico* web-based applications predicted this variant to be disease-causing by MutationTaster (25) and damaging by SIFT (sorts intolerant from tolerant) (26). We studied all five healthy siblings of our patient for the this variant, and none was homozygous (confirming appropriate segregation within the family (Figures 1A,B). The variant was classified variant of uncertain significance (VUS) according to ACMG classification (PM2, PP3, PP4) (27). To better understand the effect of the variant, we used a structural approach, as the crystal structure of GARP in complex with LAP is available (PDB ID 6GFF). The p.327Ile > Thr is likely to destabilize the LRR structure, both by introducing a polar residue into a hydrophobic pocket and by, at the same time, introducing an unoccupied cavity that destabilizes the structure. Such disturbance might not be reflected in protein expression levels but can still result in GARP aggregation and loss of function, resulting in partial mature TGF-β inactivation. However, the proposed mechanism for a missense variant leading to loss of function effect would require further functional validation. Taken together, the comparable, highly distinctive phenotype seen in our patient and in the previously reported patients, in addition to the supporting evidence, suggest that the identified variant is involved in our patient’s phenotype.

**TABLE 1:** Clinical and genetic characteristics of patients with cleft palate and retinopathy syndrome associated with LRRC32 pathogenic variants.

	Family A, Individual III-3	Family A, Individual III-4	Family B, Individual III-1	Current Patient
Age at last exam	3 years–2 months	2 years–11 months	3 years–3 months	14years–6 months
Gender	Female	Male	Male	Male
Gestational age	40 weeks + 3 days	34 weeks	36 weeks	34 weeks
Birth weight (Z score)	2,380 g (–2.56)	1,340 g (–2.2)	1,740 g (–2.81)	1,170 g (–2.6)
Global developmental delay	+	+	+	+
Developmental quotient	72	57	23	65
Age at walking	24 months	27 months	Non-ambulatory	30 months
Speech delay	+	+	+	+
Height (Z score)	88.5 cm (–1.74)	86 cm (–2.39)	90 cm (–1.89)	135 cm (–3.41)
Weight (Z score)	13.2 kg (–0.65)	12.5 kg (–1.23)	12 kg (–2.03)	25 kg (–4.25)
Head circumference (Z score)	48.2 cm (–0.3)	47 cm (–1.6)	46.8 cm (–1.7)	46.8 cm (–2.1)
Dysmorphic features	Midface retrusion	Micrognathia	Short philtrum	Triangular face, micrognathia, posteriorly rotated ears, high protruding nasal bridge
Hypotonia	+	+	+(axial hypotonia, increased peripheral tone)	+
Atopic dermatitis	+	+	NA	no
Cleft palate	+	+	+	+
Myopia	+	+	+	–
Retinopathy	Proliferative retinopathy	Proliferative retinopathy	Proliferative retinopathy	Proliferative vitreoretinopathy
Strabismus	–	+	–	–
Hearing loss	Mild-moderate	Mild-moderate	–	Mild-moderate
Brain MRI	Cavum septum pellucidum, mild ventriculomegaly	Ventriculomegaly	Ventriculomegaly, partial agenesis corpus callosum, vermian hypoplasia	Decreased white matter volume in temporal and right parietal areas, prolonged relaxation time in temporal and subcortical regions
Echocardiography	NA	Normal	Normal	Normal
Sleep apnea	+	NA	+	–
Pathogenic variant in LRRC32 gene	c.1630C > T p.Arg544Ter	c.1630C > T p.Arg544Ter	c.1630C > T p.Arg544Ter	c.980T > C p.Ile327Thr
References	(5)	(5)	(5)	Current

*MRI, magnetic resonance imaging; NA, not available; g, grams; kg, kilograms.*

The novel syndrome of GARP deficiency is characterized by the combination of cleft palate, progressive (childhood onset) retinopathy, developmental delay and growth retardation. Table 1 summarizes the clinical, radiological and genetic features of all four cases reported so far. Our work somewhat expands the clinical spectrum of this new syndrome. In particular the proliferative retinopathy seen in our patient was typically located in the vitreoretinal region and its progression required laser photocoagulation. Furthermore, we show that growth retardation may be more severe than previously reported. Previous patients had modest decrease of height and weight z-scores, whereas our patient had severe restriction of both his height and weight (Table 1). In addition, we emphasize the associated facial dysmorphism to be dominated by triangular face, micrognathia and high protruding nasal bridge. Finally, MRI changes in our patient showed decreased white matter volume in contrast with ventriculomegaly and corpus callosum abnormalities that were seen in the previously reported patients.

The common phenotype described in all four patients with the discussed syndrome (Table 1) is consistent with the impairment of some aspects of TGF-β function. This strongly supports the involvement of the GARP mutation by impairment in TGF-β maturation. In conclusion, loss of function bi-allelic pathogenic variants in *LRRC32* should be considered as associated with a distinctive cleft palate-retinopathy syndrome.

Finally, the introduction in recent years of next generation sequencing technologies (whole exome sequencing and genetic panels) into clinical practice has enabled successful resolution of several undiagnosed genetic cases in various clinical fields. Relevant to this report, and given the genetic heterogeneity of clefting syndromes, we strongly suggest to include *LRRC32* in gene panels targeted for diagnosis of patients with cleft palate.

## Data Availability Statement

The data generated or analyzed during this study are included in this published article. The *LRRC32* variant was submitted to ClinVar (accession no. VCV001330304.1).

## Ethics Statement

The studies involving human participants were reviewed and approved by the EMC-0067-09 Emek Medical Center. Written informed consent to participate in this study was provided by the participants’ legal guardian/next of kin.

## Author Contributions

RS led the composition and evaluation of the manuscript, designed and conceptualized the study, and interpreted the data. ZH-E and YZ wrote the draft and interpreted the clinical data. BF designed and conceptualized the study. HJ-H analyzed and interpreted the ophthalmological studies. MD and LI-T evaluated and analyzed the protein structural analysis. OE analyzed and interpreted the genetic data, in particular the exome sequencing. MK analyzed and interpreted the *LRRC32* variant in the family and reviewed and evaluated the manuscript for content. All authors reviewed the final draft before submission.

## Conflict of Interest

The authors declare that the research was conducted in the absence of any commercial or financial relationships that could be construed as a potential conflict of interest.

## Publisher’s Note

All claims expressed in this article are solely those of the authors and do not necessarily represent those of their affiliated organizations, or those of the publisher, the editors and the reviewers. Any product that may be evaluated in this article, or claim that may be made by its manufacturer, is not guaranteed or endorsed by the publisher.

## Footnotes

1
https://gnomad.broadinstitute.org
